# Relationship of Different Properties from Non-Destructive Testing of Heavy Concrete from Magnetite and Serpentinite

**DOI:** 10.3390/ma14154288

**Published:** 2021-07-31

**Authors:** Petr Lehner, Jacek Gołaszewski

**Affiliations:** 1 Department of Structural Mechanics, Faculty of Civil Engineering, VSB-Technical University of Ostrava, Ludvíka Podéště 1875/17, 708 33 Ostrava-Poruba, Czech Republic; 2 Department of Building Processes and Building Physics, Faculty of Civil Engineering, Silesian University of Technology, Akademicka Street 5, 44-100 Gliwice, Poland; jacek.golaszewski@polsl.pl

**Keywords:** magnetite aggregate, Portland cement, radiation-shielding concrete, self-compacting concrete, serpentine aggregate, slag cement, diffusion, NDT

## Abstract

Radiation-shielding concrete has been analyzed by several methods of destructive and non-destructive testing (NDT). Concretes made of crushed basalt, magnetite, serpentinite, and two different types of cement (Portland cement CEM I and slag cement CEM III/A) were studied. In this study, we analyzed concrete columns with a height of 1200 mm and a cross-section of 200 × 200 mm^2^. The top and bottom of the column were analyzed using data from compressive strength, dynamic modulus of elasticity, water penetration, and diffusion coefficients derived from the electrical resistivity test. This article presents the properties of fresh concrete and concrete after two years of setting. It was determined how the different ratios of basalt, magnetite, and serpentinite affect the individual measured parameters. Furthermore, correlation relations between individual resulting values were analyzed. It was observed that compressive strength generally does not correlate with other results. The diffusion coefficient correlated well with density and water penetration. Little or no correlation was observed in the diffusion coefficient with compressive strength and modulus of elasticity. The results of the study make it possible to refine the testing of heavy concretes in terms of electrical resistivity, and point to the possible use of NDT methods. The results also show which composition of heavy concrete is better in terms of effective diffusivity.

## 1. Introduction

The use of nuclear energy instead of burning fossil fuels is an ever-expanding process. New nuclear power plants are under construction around the world, and require high-quality building materials [1]. Research in the field of radiation-shielding concrete structures for nuclear power plants or laboratory equipment is on the rise in this spirit. Depending on the different types of radiation and energy spectra, different materials are used [2], and the research into heavy concrete shielded against radiation leads to many approaches. Some studies show chromium-ore-based concretes containing various types of minerals [3] or other additives [4,5,6], as well as considering their ecological and sustainability aspects, such as in terms of recycling [7]. Others examine magnetite and serpentinite concretes [8,9,10], and their advantages and disadvantages.

The dynamic [11,12] and thermodynamic properties [13] of concrete have also been investigated, and various numerical models have been prepared [14,15]. In general, it is necessary to evaluate both the production intensity and the sustainability of various concrete mixtures [16,17,18]. From this point of view, it is desirable to improve non-destructive testing (NDT) methods for testing heavy concretes, and to evaluate which NDT methods are replaceable, or whether it is possible to use them as a supplement to standard tests. 

The general requirements for the protection of concrete against radiation are high density, low porosity, and good workability [19]. The preparation of high-quality heavy concrete carries many difficulties that need to be researched in the long term [20]. In the first part of the research, the basic properties of heavy self-compacting concrete (SCC) mixtures were investigated, as well as the effect of high weight on formwork [10]. These concretes have been shown to have good radiation-shielding properties, and the proposed formwork solution has shown to be sufficiently load-bearing and stable. As a large amount of reinforcement is generally used in protective concrete structures, and it is also logically assumed that the structure will be exposed to other aggressive substances, it is necessary to examine the mixture in terms of diffusivity [21,22].

Diffusion, and thus resistance to penetration, is also significantly affected by cracks and microcracks [23,24], which needs to be assessed by long-term evaluation and measurement of this special concrete. Heavy concrete during its preparation can be analyzed via destructive methods, but when involving it in the structure, it is necessary to think about evaluation using non-destructive testing (NDT) methods. This research aims to present the results of measurements on concrete columns several years after concreting. These measurements are of mechanical properties and properties related to water penetration and diffusion. All presented results make it possible to scale the prepared concretes and evaluate their advantages and disadvantages. Furthermore, the comparison of methods certainly allows the substitutability of NDT methods or, conversely, the indispensability of destructive methods. The results will help in the preparation of new heavy concrete mixtures, and also in the necessary long-term analysis of existing structures in places with hazardous radiation.

## 2. Materials and Methods

### 2.1. Materials and Mix Design

Our choice of materials for radiation-shielding concrete was based on a published study [10]. The choice of special aggregate—i.e., magnetite and serpentinite in different fractions—was governed by its basic properties; namely, the high atomic number. Another suitable property of these aggregates is their high water binding, which is favorable for radiation protection. Basalt, which is commonly used, as well as quartz sand (density of 2.65 kg/dm^3^), were chosen as supplementary aggregates. The chemical compositions and properties of types of cement are listed in Table 1.

Another important factor is the choice of cement. Two types of cement were selected for the study [10]—namely, Portland cement CEM I 42.5 N, and slag cement CEM III/A 42.5 N, following EN 197-1 [25]. These two types of cement have high resistance to initial cracking and long durability, due to low heat of hydration, resistance to sulfates, and low alkali content [26]. The basic chemical composition and properties of each type of cement are given in Table 2.

The analyzed concrete mixtures also contained a superplasticizer based on polycarboxylate (HRWR) and water [27]. Another admixture was viscosity-modifying admixture (VMA), which helped the stability of the two blends [28]. The composition of the mixtures is given in Table 3. It can be seen that the ratio of water to cement (*w*/*c*) was 0.48 for all mixtures except one, where it was 0.60. For this mixture, however, the effective ratio was 0.48. The selection of fractions was first-order governed by ratios of magnetite and serpentinite of 2:1 and 1:2, and was further supplemented so that the sand fully complemented all fractions [29]. The goal was a slump flow of 600 mm while maintaining the highest possible density of the mixture.

### 2.2. Preparation of Specimens 

**Figure 1:**
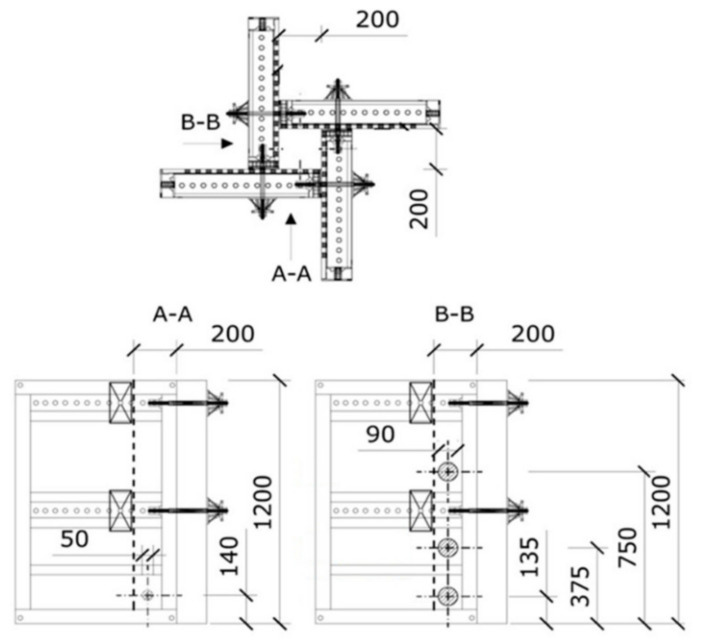
Schematic drawing of column formwork (size in mm) [10].

In addition to the standard samples used for the elementary tests, columns with a height of 1200 mm and a cross-section of 200 × 200 mm^2^ were prepared for the long-term measurement of rheological properties (the formwork scheme of the column is shown in Figure 1). The columns were demoulded after 3 days and then stored in laboratory conditions at a temperature of 20 °C and 40% humidity for two years. The results of the elementary tests and the rheological properties were deeply evaluated in an already-published article [10].

**Figure 2:**
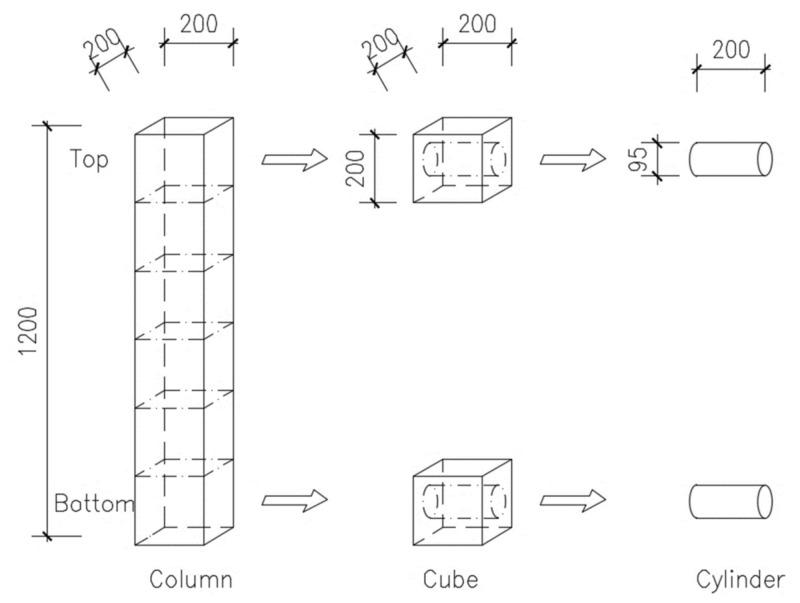
Schematic of cutting sections of columns to obtain individual cubes, and subsequent boreholes to obtain cylinders (size in mm).

For further testing, the columns after two years of solidification were cut into several cubes vertically (see diagram in Figure 2). The cubes in the middle were used to test the compressive strength and modulus of elasticity, while the top and bottom parts were drilled to obtain cylinders. These were then analyzed, first at ultrasonic speed to obtain the dynamic moduli of elasticity, and then using electrical resistivity to obtain the diffusion coefficients. The intention was to check whether the properties at the bottom and top of the columns were the same or different.

### 2.3. Testing Methods

At the beginning of the laboratory investigation, individual concrete mixtures were prepared, and the basic properties of fresh concrete were analyzed. Further testing was also performed on samples cut from the columns two years after concreting, in order to evaluate the effects of long-term curing of the concrete.

#### 2.3.1. Air Content and Density

According to EN 12350-7 [30] and EN 12350-6 [31], the air content and density of concrete were determined. After two years, when the columns were cut into individual cubes, the density was again analyzed, mainly in terms of comparing the resulting values in the top and bottom parts of the columns.

The bulk density of concrete was determined in the naturally wet state as the ratio of the weight of a given amount of hardened concrete to its volume. Subsequently, it was possible to determine whether the concretes were light (up to 2000 kg/m^3^), standard (2000–2600 kg/m^3^), or heavy (above 2600 kg/m^3^).

#### 2.3.2. Compressive Strength and Water Penetration

Compressive strength was tested according to EN 12390-3 [32] on cubic samples cut from columns. The test specimens were carefully ground, and the compressive strength of each test specimen was further determined by dividing the maximum load by the cross-sectional area calculated from the mean diameter. According to the standard, the areas of command were also inspected.

The depth of water penetration was also determined in cubes cut from columns at the age of two years, in accordance with EN 12390-8 [33]. Measurement of water penetration was important for estimating the watertightness of concrete, and for evaluating the possible effects of mixture segregation.

#### 2.3.3. Dynamic Modulus of Elasticity

An ultrasonic pulse method was used to determine the dynamic modulus of elasticity according to EN 12504-4 [34]. This method is based on the repeated transmission of ultrasound pulses to the tested material and the determination of the transit time of the ultrasound pulse.

The dynamic modulus of elasticity was determined from the bulk density of concrete and the time of ultrasound passage through the sample. The test was performed on cylindrical samples from the top and bottom sections of columns. These samples had a diameter of about 95 mm and a length of about 200 mm.

#### 2.3.4. Electrical Resistivity and Diffusion Coefficient

A Wenner probe (manufactured by Proceq, Schwerzenbach, Switzerland) was used to measure the electrical resistance of the surface. The test was performed on cylindrical samples from the top and bottom parts of columns according to AASHTO T358 standards [35]. Surface resistance was measured from four longitudinal sides, and then the mean value and standard deviation were determined. In addition, the specific resistivity is a method of indirect measurement of the degree of diffusion of chloride ions [16]. The diffusion coefficient of chloride ingress was determined from the determined values of electrical resistivity using the Nernst–Einstein equation [36].

It is necessary to note that, in principle, this process is an NDT method. However, in this case, the drilled cores were analyzed. To maintain the principles of NDT, it would be possible to evaluate the surface resistivity directly on the construction of columns without the need for drilling. However, the cores were drilled earlier for other tests.

It should be noted that many other properties of the presented concretes have already been published [10]—especially the basic values for fresh concrete.

## 3. Results

As shown in Figure 2, two samples from one column were analyzed: the top and bottom parts. Therefore, the results for individual concrete mixtures are marked with the letters T and B. Thanks to this, it is possible to study the effect of self-compaction on the formwork in the results. It is, of course, necessary to consider the load of the lower part of the column by its weight, as the measurement took place 2 years after concreting. From this point of view, the bottom part was expected to have worse properties. On the other hand, the effect of self-compaction can affect the homogeneity and heterogeneity of concrete at different levels.

### 3.1. Values According to the Mixture

**Figure 3:**
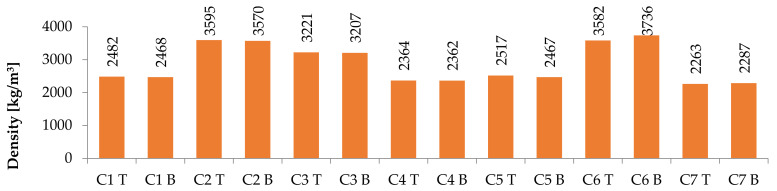
Density results at the top (T) and bottom (B) parts of columns for individual concrete mixtures.

The first parameter of measurement was density (see Figure 3). The difference for all mixtures between the top and bottom parts did not exceed 1.5%, showing that there was no problem with segregation. Concretes that do not contain magnetite 0/16 showed a density of around 2500 kg/m^3^. On the other hand, concretes C2, C3, and C6, which do contain magnetite 0/16, showed a high density, up to 3600 kg/m^3^. The type of cement did not affect the result. This is in line with expectations.

**Figure 4:**
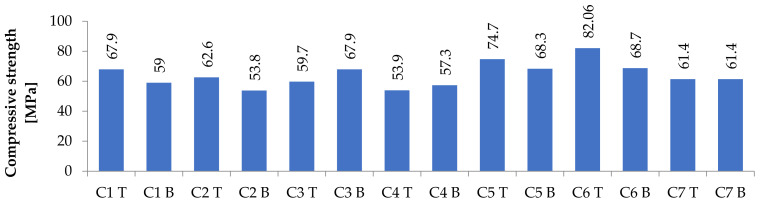
Compressive strength results at the top (T) and bottom (B) parts of columns for individual concrete mixtures.

Another result was compressive strength (see Figure 4). As addressed above, the strength of the bottom part could be expected to be less than that of the top part. This was confirmed for all concretes except for C3 and C4. The differences for other concretes were between 10 and 15%. Concretes where CEM I was used (C1, C2, C3, and C4) had slightly lower values than concretes where CEM III/A was used, due to CEM III/A long-term curing characteristics. The lowest average strength was achieved by concrete, where magnetite 0/5, serpentinite 2/8, and serpentinite 8/16 were used.

**Figure 5:**
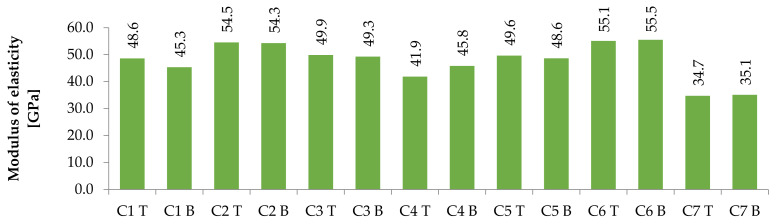
Modulus of elasticity results at the top (T) and bottom (B) parts of columns for individual concrete mixtures.

The results of the modulus of elasticity showed very similar behaviour in terms of comparison between the mixtures as the compressive strength did (see Figure 5). Here, again, most of the concrete had a lower value at the bottom, but the differences ranged from 1% to 9%. Due to the nature of ultrasonic measurements, these deviations are negligible. The influence of the type of cement was unprovable. Again, the composition of the aggregates played an important role. Concretes C2 and C6, which contain magnetite 0/5 and magnetite 0/16, respectively, showed the best properties.

**Figure 6:**
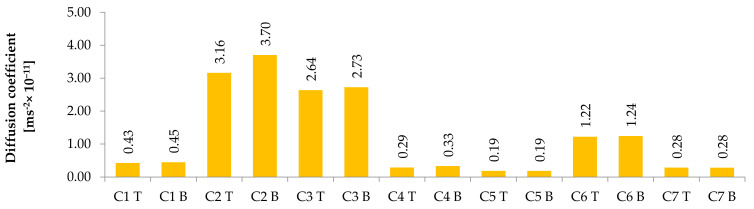
Diffusion coefficient results at the top (T) and bottom (B) parts of columns for individual concrete mixtures.

The last—but no less important—result was the apparent diffusion coefficient (see Figure 6). This is a parameter related to the penetration of chlorides and other aggressive substances. This parameter can be used to compare a set of concrete mixtures with one another, but also as an input parameter for modelling chloride diffusion.

The differences between the top and bottom parts of the columns were insignificant in terms of measurement tolerance. On the other hand, the significance of the differences between the individual concretes was high. Concretes containing mainly 2/16 basalt or serpentinites showed low diffusion coefficient values, while concretes containing magnetite showed high diffusion coefficient values. Of particular interest are the lower values of C6 concrete, which is made of magnetite but contains CEM III/A cement. It is necessary to make clear that in terms of resistance, a low diffusion coefficient is better than a high one. Thus, the evaluation of concretes C2 and C3 shows that they have less resistance than other concretes.

The resistance of CEM III/A cement concrete to water penetration in the direction perpendicular to the concreting was higher than that of CEM I cement concrete. The water penetration results are shown in Table 4.

### 3.2. Correlation with Diffusion Coefficient

**Figure 7:**
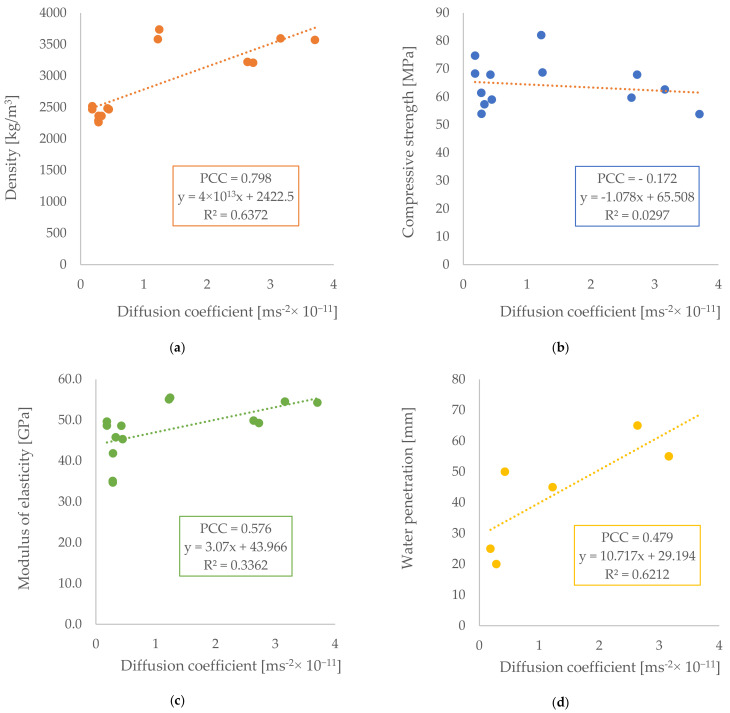
Correlation between the diffusion coefficient and: (**a**) density, (**b**) compressive strength, (**c**) modulus of elasticity, and (**d**) water penetration.

The significant advantage of measuring resistivity is that it is a form of NDT. Because of that, the results of all other tests were compared with the results of the diffusion coefficient with regard to potential relationships. In Figure 7, the relationships of the linear regression of the diffusion coefficient with density, compressive strength, modulus of elasticity, and water penetration can be seen. Pearson’s correlation coefficient (PCC) was obtained. It is possible to observe correlations between the diffusion and density parameters, and between the diffusion and water permeability parameters. In both cases, the increase in the diffusion coefficient is related to the increase in the parameters of density and water penetration. There is a small correlation with the modulus of elasticity, and no correlation with compressive strength.

### 3.3. Correlation of Other Parameters

**Figure 8:**
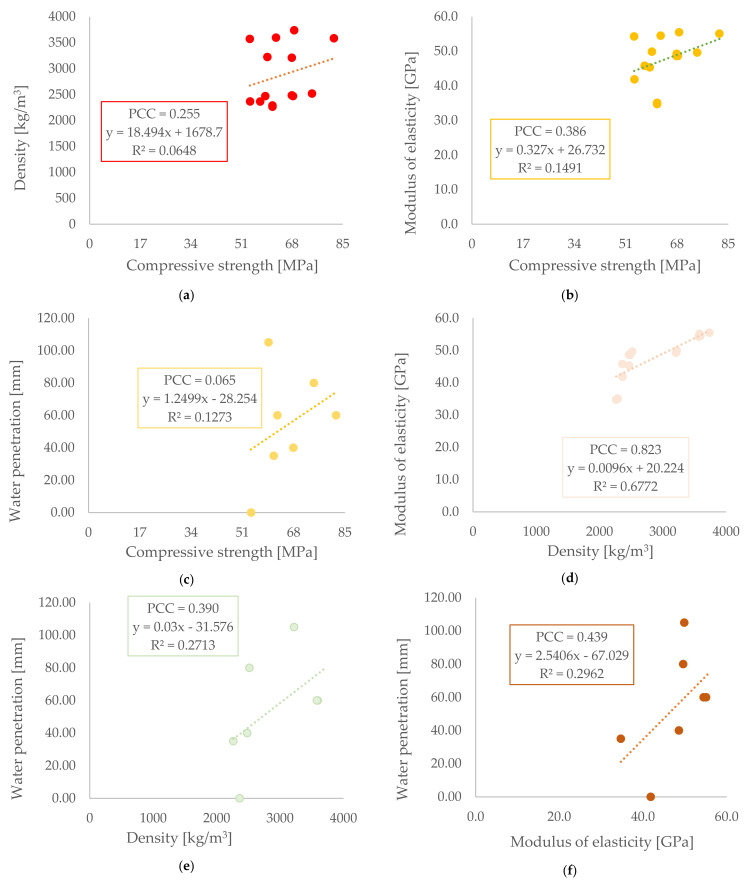
Correlation between (**a**) compressive strength and density, (**b**) compressive strength and modulus of elasticity, (**c**) compressive strength and water penetration, (**d**) density and modulus of elasticity, (**e**) density and water penetration, and (**f**) modulus of elasticity and water penetration.

To supplement the results, correlations between all measured and presented values were also evaluated. The individual parameters were density, compressive strength, modulus of elasticity, and water penetration. By comparing all of them, six pairs were created. The results are shown in Figure 8.

No correlation was observed between the individual parameters, except for between density and modulus of elasticity. Interestingly, all values showed an upward trend. The only graph where the trend is declining is between the diffusion coefficient and the compressive strength.

## 4. Discussion

Previously, significant differences in the permeability and moisture diffusion coefficients of heavy concretes, depending on the location of the samples, were found [37]. However, the present results show that in the case of a well-designed SCC mixture, this is not a problem, and the values from the top and bottom parts of the columns are very similar. Differences in the compressive strength of concrete in the cut specimens were observed, as different curing rates are expected, associated with the types of cement used. After long-term hardening of the columns, it has been shown that the strength of the concrete at the top is higher in most columns. On the other hand, the values at the bottom were sufficient. Values over 50 MPa can be considered adequate for the requirements and conclusions of other heavy concrete studies [38,39].

The modulus of elasticity was slightly different. The difference between the upper and lower parts was negligible, and concrete containing magnetite was shown to achieve the best results. Conversely, the worst was concrete with serpentinites of all fractions. As stated in a study of high-strength concretes [40], the evaluation of the modulus of elasticity according to the standards may be underestimated. Even from this point of view, values higher than 40 GPa are very good.

Diffusion parameters showed significant differences between concretes. It should be emphasized that the values were obtained by measuring the electrical resistance, which is affected by the conductivity properties of the material, porosity, etc. [41]. Therefore, these results need to be taken with caution, and the study of heavy concretes must be deeply expanded in the future, as other comparable data are lacking. All used aggregates should have similar conductivity, but have different water penetration. The results of the comparison of diffusion coefficients with other measured parameters thus showed that there is a higher correlation with water penetration, and also with density, but on the other hand, there is little or no correlation with the modulus of elasticity, or with compressive strength. A comparison of the results of the other parameters did not show this correlation; only for the modulus of elasticity and density was the correlation observed. This may be due to the principle of determining the modulus of elasticity using the ultrasonic velocity, which is related to density but not directly dependent on it [42]. It should be noted that these results need to be supplemented by radiation resistance, which is the most fundamental result.

## 5. Conclusions

This article presented the results of measurements on samples obtained from columns of heavy concrete two years after concreting. These heavy concretes were prepared with the main requirement being radiation resistance. The differences between the top and bottom parts of the columns were evaluated, and the individual results were also compared in order to analyze the similarities. The following conclusions were reached:

1. The best results in almost all tests were shown by C2 concrete, which contains about 30% magnetite 0/5 and about 70% magnetite 0/16. Conversely, in the evaluation of the diffusion coefficient, this concrete was the worst;
2. The worst results in almost all tests were shown by C7 concrete, which contains ~20% serpentinite 0/2, ~60% serpentinite 2/8, and ~20% serpentinite 8/16;
3. A correlation was observed between the diffusion coefficient and the density, between the diffusion coefficient and the water penetration, and between the density and the modulus of elasticity;
4. Evaluation of heavy concretes using electrical resistivity must be further expanded by a larger number of samples and ratios of aggregates in concrete mixtures.

These results can help to expand knowledge about heavy concretes and their evaluation using destructive and non-destructive methods. Ultrasonic testing and electrical resistivity testing as NDT methods may be suitable for the analysis of existing structures.

## Figures and Tables

**Table 1:** Chemical compositions, physical properties, and strength of both types of cement [10].

Chemical Composition and Properties	CEM I 42.5N	CEM III/A 42.5N
SiO_2_ (%)	21.48	31.38
Al_2_O_3_ (%)	4.80	5.98
Fe_2_O_3_ (%)	2.62	2.09
CaO (%)	65.60	52.51
MgO (%)	0.87	3.73
SO_3_ (%)	2.84	1.45
K_2_O (%)	0.47	0.56
Na_2_O (%)	0.12	0.34
Cl (%)	0.008	0.058
Loss on ignition (%)	1.12	0.12
Flow (cm)	18.1	15.4
Water demand (%)	28.0	34.0
Blaine (cm^2^/g)	3800	4700
Density (g/cm^3^)	3.15	2.99
28-day bending strength (MPa)	8.1	9.5
28-day compressive strength (MPa)	52.6	58.2

**Table 2:** The properties of the crushed aggregates [10].

Type of Aggregate *	Density (kg/m^3^)	Water Absorption (%)
Crushed basalt 2/16	3000	0.80
Crushed serpentine 0/2	2600	2.14
Crushed serpentine 2/8	2600	2.41
Crushed serpentine 8/16	2600	1.47
Crushed magnetite 0/5	4800	0.40
Crushed magnetite 0/16	4800	0.40

* Numerical values indicate aggregate fractions (mm).

**Table 3:** Concrete mixtures compositions and properties.

Composition and Properties	C1	C2	C3	C4	C5	C6	C7
Cement CEM I (kg/m^3^)	350	350	350	350	-	-	-
Cement CEM III (kg/m^3^)	-	-	-	-	350	350	350
Water (kg/m^3^)	168	168	168	168	168	168	211
*w*/*c*	0.48	0.48	0.48	0.48	0.48	0.48	0.60
*w*/*c*_eff_	-	-	-	-	-	-	0.48
Quartz sand 0/2 (kg/m^3^)	687	371	371	371	687	371	371
Crushed basalt 2/16 (kg/m^3^)	1001	-	-	-	1001	-	-
Crushed magnetite 0/5 (kg/m^3^)	300	839	772	895	300	839	-
Crushed magnetite 0/16 (kg/m^3^)	-	1846	1018	-	-	1846	-
Crushed serpentine 0/2 (kg/m^3^)	-	-	-	-	-	-	273
Crushed serpentine 2/8 (kg/m^3^)	-	-	485	485	-	-	909
Crushed serpentine 8/16 (kg/m^3^)	-	-	371	485	-	-	273
HRWR CEM I (%m.c.)	0.36	0.3	2	1.6	-	-	-
HRWR CEM III/A (%m.c.)	-	-	-	-	0.2	0.2	1.4
VMA (%m.c.)	0.15	-	-	-	-	-	-
Expected density (kg/m^3^)	2506	3574	3537	2756	2506	3574	2389

**Table 4:** Water penetration resistance of the concrete.

Composition and Properties	C1	C2	C3	C4	C5	C6	C7
Water penetration (mm)	40	60	105	n/a *	80	60	35

* Water penetrated through the sample.

## Data Availability

All necessary data are presented in the article.
